# Spontaneous Hybridization between *Pinus mugo* and *Pinus sylvestris* at the Lithuanian Seaside: A Morphological Survey

**DOI:** 10.1100/2012/172407

**Published:** 2012-04-24

**Authors:** Darius Danusevičius, Vitas Marozas, Gediminas Brazaitis, Raimundas Petrokas, Knud Ib Christensen

**Affiliations:** ^1^Faculty of Forestry and Ecology, Aleksandras Stulginskis University, Studentu Street 11, Kaunas region, 53361 Akademija, Lithuania; ^2^Institute of Forestry, Lithuanian Research Centre for Agriculture and Forestry, Liepu Street 1, Girionys, 53101 Kaunas, Lithuania; ^3^c/o Botanical Garden, Natural History Museum of Denmark, University of Copenhagen, Oster Farimagsgade 2B, 1353 Copenhagen, Denmark

## Abstract

We address the problem of spontaneous hybridization between an exotic species *Pinus mugo* and the native/local *P. sylvestris* at the seaside spit of Kursiu Nerija in Lithuania. The objective was to identify spontaneous hybrids between *P. mugo* and *P. sylvestris* based on morphology traits among the individuals naturally regenerating at the seaside spit. The field inventory was carried out over the entire Lithuanian part of the spit, and 200 individuals morphologically intermediate between *P. sylvestris* and *P. mugo* were identified. Based on a weighted trait index, the intermediate individuals were grouped into two groups, one morphologically close to *P. sylvestris* and another close to *P. mugo*. The needle micromorphological traits of the putative hybrids were of intermediate values between *P. mugo* and *P. sylvestris*. The results provide a strong evidence of spontaneous hybridization between *P. mugo* and *P. sylvestris* in Lithuanian seaside spit of Kursiu Nerija.

## 1. Introduction

*Pinus mugo* Turra is an exotic forest tree species in Lithuania introduced to the Lithuanian seacoast spit of Kursiu Nerija (Curonian Spit, or further abbreviated as Nerija) in approximately 1820 to prevent sand erosion by establishing a forest cover [1, 2]. At present, *Pinus mugo* has reached its natural maturity and variable options were presented regarding its future. Kursiu Nerija represents a unique environment granted the status of the UNESCO World heritage site. In this study, we address the problem of spontaneous hybridization between *P. mugo* and the local *P. sylvestris* in Nerija as a possible contribution in solving the *P. mugo* replacement issue. From the theoretical point of view, it may provide an important knowledge for the studies of species divergence and speciation with the material introduced over 200 years ago. Similar introductions with interbreeding species may exist elsewhere, and our study may draw the attention to positive and negative evolutionary consequences of hybridization and introgression between species in forested landscapes.

In the Alps, putative hybrids between *P. mugo *and* P. sylvestris* were observed already in the 19th century and named *P.* x *rhaetica* Brügger [3]. Since then, various morphological and anatomical characters have been used to verify spontaneous *P. mugo *× *sylvestris* in the autochthonous populations of its parents in Pyrenees, Carpathians, Tatra Mountains, and Alps [1, 4–7] as well as in planted stands of *P. mugo* and *P. sylvestris* in Denmark [8]. A number of biochemical marker-based studies have also successfully identified the *P. mugo *×* P. sylvestris* hybrids [9, 10]. Recently, the paternally inherited chloroplast DNA markers were developed and tested on artificial crosses between *P. mugo* and *P. sylvestris* for identification of the male parent of the suspected hybrids [6, 11, 12]. All these studies provide solid evidence of *P. sylvestris *×* P. mugo* hybridization and introgression and support for the probability of the spontaneous hybridization in Lithuanian Nerija, where the naturalized *P. mugo* grows together with both indigenous and planted stands of *P. sylvestris*.

*P. mugo* is native to the mountains of central and southern Europe and is a morphologically highly variable species, and there currently is no consensus on how it should be treated taxonomically (review by [13]). High morphological polymorphism, the adaptation to specific adaptive environments, and high intra-taxon hybridization rates make the taxonomic subdivision difficult. Being geneticists and a taxonomist advocating a broad species concept, we use the taxonomic approach suggested by [5]: one species *P. mugo* (Turra) divided into three subspecies: *Pinus mugo* subsp. *mugo*, *Pinus mugo* subsp. *uncinata* (Ramond) Domin, and the nothosubspecies *Pinus mugo* nothosubsp. × *rotundata* (Link) Janch. and H. Neumayer (a hybrid taxon between *P. mugo* subsp. *uncinata* × *P. mugo *subsp. *mugo*). *Pinus mugo* subsp. *uncinata* is usually a monocormic tree reaching the height of 14–20 meters with asymmetrical cones having hooked apophysis and is native to subalpine zone up to tree limit and is more common in the western part of the range of *P. mugo* s.lat. (Iberian peninsula, [13]). There are presumptions that *P. mugo* subsp. *uncinata* may have shared genome with *P. sylvestris* in its evolutionary past [8, 13]. *P. mugo* subsp. *mugo* is a polycormic shrub up to 1.5 meter tall with prostrate growth, symmetrical cones with flat apophasis and is found in the mountains of the central and the eastern parts of the species range. *P. mugo *subsp*. rotundata* possesses an intermediate morphology between the subspecies *uncinata* and *mugo *as is common in the peat bogs at the central part of the range.

In 1768, professor Johann Daniel Titius from University of Wittenberg was the first person to suggest afforesting sand dunes with conifer forests in Denmark. Following the professor's initiative, *P. mugo *subsp*. mugo *was introduced in 1798 and *P. mugo* subsp. *uncinata* in 1886 to afforest sandy dunes in the north-western part of Denmark ([8] and references therein). *P. mugo* was introduced to the Lithuanian seacoast spit of Kursiu Nerija in 1825, after seven villages were destroyed by moving sands. Kursiu Nerija is a narrow strip of sand stretching 97 kilometres along the Baltic Sea in western Lithuania. An East Prussian forester Georg David Kuwert in cooperation with a Danish environmentalist Bjørn Sørensen initiated an afforestation programme with *P. mugo* starting at the southern part of the Nerija spit in Lithuania [2, 14]. Since then *P. mugo *has been planted on the seaside dunes, where it is well adapted and often naturalized. In 1904, the large-scale afforestation programme was completed with most of the dunes covered with forests and the eroding dunes were stabilized [2, 14].

Since its introduction to Nerija, the morphological variation of the exotic* P. mugo* and putative hybrids with the local *P. sylvestris* has never been studied. The objective of our study was to identify spontaneous hybrids between *P. mugo* and *P. sylvestris *based on morphological traits among the individuals naturally regenerating at the Lithuanian seaside spit of Kursiu Nerija. The fundamental interest is to investigate how a transferred exotic species may affect the species differentiation events with the local material. In comparison with the earlier *P. mugo *×* sylvestris* hybridization studies, the current study shows that moving *P. mugo* material to a new environment has lead to altered flowering phenology and extensive hybridization.

## 2. Material and Methods

**Figure 1:**
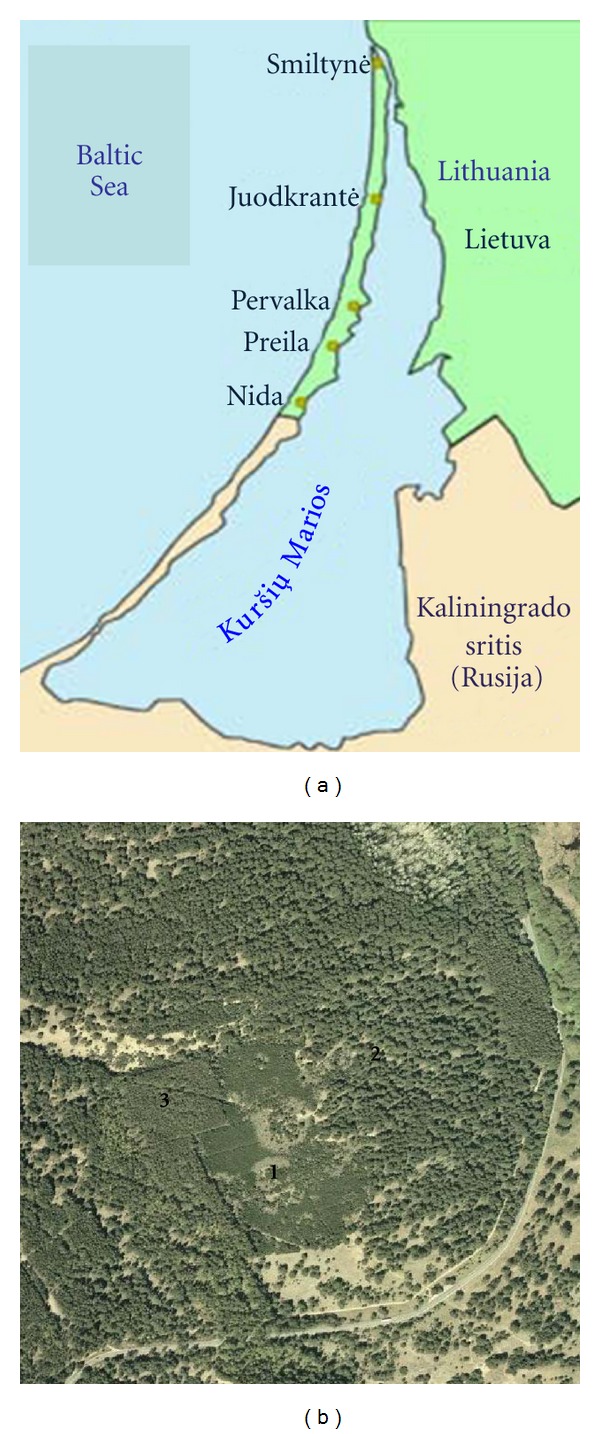
(a) Map of Kursiu Nerija spit in the seaside Lithuania with the sampling locations. (b) Arial photo of a sampling location “Avino Ragas” near Juodkrantė. *P. mugo* stand (1) is surrounded by naturally (2) and artificially (3) established *P. sylvestris *stands. The putative hybrids were sampled at the edge of the *P. mugo* stand. The introduced *P. sylvestris *may differ in flowering time from the local stand and so create more variation in flowering time and success of mating with *P. mugo*.

The field inventory of the putative hybrids was carried out in September 2010 and May 2011 in the zones of potential hybridization on the forested dunes of Kursiu Nerija spit (Figure 1(a)). The inventory was carried out in six locations representing the entire Lithuanian part of the spit (Figure 1(a); Table 1). Hybrid individuals were found as (a) naturally regenerating young trees established at the edge of large stands of *P. mugo* (presumed pollen donors) mixed with several mature *P. sylvestris* trees (presumed mother trees), (b) naturally regenerating young trees in between the forest stands of *P. mugo *and* P. Sylvestris*, and (c) mainly adult singular trees with an intermediate morphology found accidentally during the inventory. Trees more or less intermediate between *P. mugo* and *P. sylvestris *were selected and their needles sampled. The needles were placed into plastic bags, and the trees were labelled and registered on a GPS. Examples of trees with more or less intermediate morphology were (a) *P. sylvestris-*like individuals with shoots, cones, bark, and needles resembling those of *P. mugo* or individuals setting cones at a young age; (b) *P. mugo* with a single stem, without bending base, grey-blue green needles (Table 2). Controls for both species were sampled.

**Figure 2:**
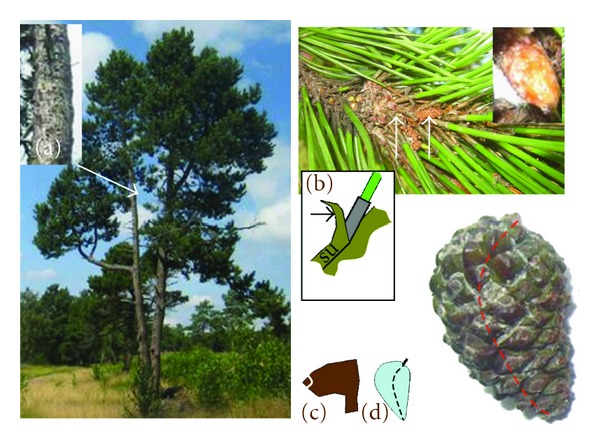
Key morphological traits of *P. mugo *subs.* uncinata.* Left: 12 meter tall tree, erect growth (resembling well *P. sylvestris, but distinguishing P. mugo *subsp.* uncinata *from* P. mugo *subsp.* rotundata and *subsp.* mugo*) needles fresh green, (a) upper stem dark grey, no reddish brawn peeling off paper flakes. Upper right: shoots dark grey with tip of bark of the stem unit prolonged and bending backwards at the base of the needle sac (b); buds are oval at the top; needles are densely attached. Lower right: cone scales are hooked (c) and cones asymmetric (d), apophysis glossy, umbo bordered.

Individuals of *P. mugo* nothosubsp. *rotundata *(including subsp.* pseudopumilio*), subsp. *mugo* were easy to separate from *P. sylvestris*. The distinction between *P. mugo *subsp.* uncinata *and* P. sylvestris *was more complicated. Like *P*. *sylvestris*, *P. mugo *subsp. *uncinata* is usually a monocormic tree up to 20 m tall and 30–40 cm thick (Figure 2). We constructed an index of key traits separating *P. mugo *subsp.* uncinata* from *P. sylvestris*, so that sum of the index values for *P. mugo *subsp*. uncinata* approaches 0 and that for *P. sylvestris* approaches 1. An intermediate value of a trait was given a score of 0.5. In this way, for each morphological trait, three types of scores were obtained: 0, 0.5, or 1 (Table 1). Index weights were added subjectively to strengthen the effect of important traits so that the index weights sum up to 1 (Table 1). The index of weighted score sum (WSS) was calculated for each potential hybrid, separately for adult and young trees as follows (abbreviations of the variables are explained in Table 2):

WSS_adult_ = 0.2 BARK + 0.3 NEEDLE + 0.1 APOP + 0.05 UMBO + 0.05 CONSYM + 0.05 TIPSU + 0.2 SHOOT + 0.05 NDENSE,
WSS_young_ = 0.1 BARK + 0.4 NEEDLE + 0.2 TIPSU + 0.2 SHOOT + 0.1 NDENSE.
WSS may vary between 0 (perfect* P. mugo *subsp.* uncinata*) and 1 (perfect *P. sylvestris*).

Based on the WSS score, the putative hybrids were subdivided into two groups (Table 3). This subdivision was used in further studies of needle micromorphology by taking into account the putative hybrid group in Table 3. In total, 47 trees and 10 needles per tree were selected for needle micro-morphology studies, among them 8 trees of putative hybrid group A (mean WSS score = 0.77 in Table 3), 11 trees of putative hybrid group B (WSS score = 0.39), 24 trees of *P. sylvestris* (WSS score = 1), and 4 trees of *P. mugo* (WSS score = 0). The needles were sampled from the midportion of an annual shoot, which was randomly selected from a lower part of the crown. In total, 470 needles were analyzed by the aid of a digital microscope. The following needle properties were assessed (Table 1): (1) number of stomata rows on the abaxial (= curvy, convex) side of the needle, (2) number of stomata rows on the adaxial (= flat) side of the needle, (3) number of stomata within the 5 mm interval in a midrow of the central portion of the needle, (4) number of stomata within the 5 mm interval in another mid-row of the central portion of the needle (the mean number of stomata of the two rows was used in the analysis). The stomata row index, known as a good discriminator between *P. mugo* and *P. sylvestris* [8], was calculated as the ratio between the numbers of abaxial and adaxial stomata rows (Table 2). The stomata row index is higher for *P. mugo* than for *P. sylvestris*, whereas the stomata number per unit area is greater in *P. sylvestris* than in *P. mugo* [8, 15, 16].

The ANOVA was used to assess the significance of the species and tree effects on the needle properties at the individual needle level by the following models:

(1) $$\begin{array}{c} Y_{ijk}=\mu +Tj+\varepsilon_{ijk},\\ Y_{ijk}=\mu +Si+Tj\left(Si\right)+\varepsilon_{ijk}, \end{array}$$

where *μ* is the total mean, *Si* is the species effect (*n* = 4, *P. sylvestris, P. mugo*, putative hybrid groups A and B, Table 3) *Tj* is the individual tree effect (*n* = 470 trees), *Tj*(*Si*) is the effect of individual tree nested within species, and *ε*_*ijk*_ is the random error.

The *Tukey least significant difference* (LSD) test in the ANOVA was used to assess significance of the pairwise differences between the species in the needle properties at the *P* = 0.05 significance level.

The Pearson product-moment correlation coefficients between the needle properties were calculated at both needle and tree mean levels. Also the correlation analysis was carried out separately for each species group.

The statistical analyses were carried out with the SAS software (PROC GLM and PROC CORR).

## 3. Results

### 3.1. The Morphological Variation

The morphology and phenology of the surveyed *P. mugo *trees varied markedly. At the time of inspection in May 2011, the variation in phenology among both *P. mugo *and* P. sylvestris *was large and maturation of *P. mugo* male strobili varied from immature to the stage of pollen dispersal. At the same occasion, mature *P. sylvestris* female strobili were noted on smaller part of the trees observed.

The following subspecies of the *P. mugo* complex [5], in which subsp. pseudopumilio is accepted] were identified: (a) *P. mugo* subsp. *uncinata*, a monocormic tree reaching height of 10–20 m, diameter 15 to 30 cm, straight to curvy stem, occasionally forked with other traits common to *P. mugo* complex fresh green needles, dark grey shoots of the previous year, dark grey bark with hard small fissures, cone scales with a well-developed hook and cones asymmetric; (b) *Pinus mugo* subsp. *rotundata*, a multicormic (occasionally monocormic) shrub with height of 4 to 6 meters, cone apophysis flat, cones are more symmetric than of *P. mugo* subsp. *uncinata*; (c) *P. mugo *subsp*. mugo*, a shrub up to 3 meters, multicormic, ascending, with symmetrical cones; (d) *P. mugo *subsp. *pseudopumilio, *a shrub with a prostrate growth 1 to 1.5 m. tall, cones with a well-developed hooked apophysis and bordered umbo. The difference between *P. mugo *subsp.* uncinata* at a younger age (lower height) and *P. mugo* subsp. *rotundata* was diffuse, because at a height of 5 to 9 meters there were trees with cone morphology resembling both subspecies.

*P. sylvestris* individuals were also variable in growth habit (sub erect to erect, polycormic to monocormic), bark colour of the upper stem, needle colour, shoot colour, cone symmetry, and development of the hook of the apophysis (see below).

In total, approximately 2000 individuals were surveyed on site during the field inventories. 200 of these bearing an unusual morphology for either *P. mugo* or *P. sylvestris* were selected for a detailed morphological examination. Of these, 23 individuals were chosen to represent the most common variants and are presented in Table 3.

Among the surveyed individuals the following deviations from the species-specific morphology were observed: (a) for *P. sylvestris*, fresh green needle colour (not greyish green), dark brown bark without papery flakes at the upper part of the stem, dark grey shoots of the previous year, cones with shinny and hooked apophysis, individuals of sub-erect growth, producing cones at a young age, ca. 5 years; (b) for *P. mugo*, straight single stem without bending at the base (rare for *rotundata* and *mugo*, but common for *uncinata*), greyish green needles, brown bark at the upper part of stem.

Based on the WSS, the individuals having atypical traits of either *P. sylvestris* or *P. mugo* were grouped into two groups depending on how close the WSS score was to the corresponding species (Table 3).

**Figure 3:**
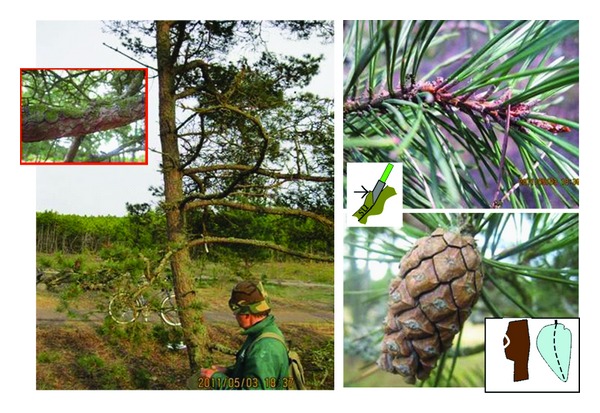
Putative *P. sylvestris* (female) × *P. mugo* (male) hybrid representing the hybrid group A (H S × M, Figure 5; Table 2; Id PD43). Left, two-stemmed, 12 meter tall (the other stem cut), bending at the base (like *mugo*); upper stem bark dark brown (*sylvestris*), no paper flakes (*mugo*) but the fissures reddish brown (*sylvestris*); upper right: shoots brown (*sylvestris*), needles greyish green (*sylvestris*), stem unit tip does not bend backwards at the needle sack (sylvestris); lower right: cone apophysis glossy (*mugo*) without hook, that is, flat (*sylvestris*), umbo bordered (*mugo*), cone symmetric (*sylvestris*).

Group A with the WSS mean of 0.84 was close to *P. sylvestris* (mean score 1), and young trees dominate in this group. The common feature to this group is the greyish-green needles and straw-coloured or brownish shoots of the previous year (both common for *P. sylvestris*) but dark brown bark on the upper stem without yellowish-brown papery flakes (except tree PR43, which had fresh green needles and light brown bark with papery flakes) (Figure 3).

**Figure 4:**
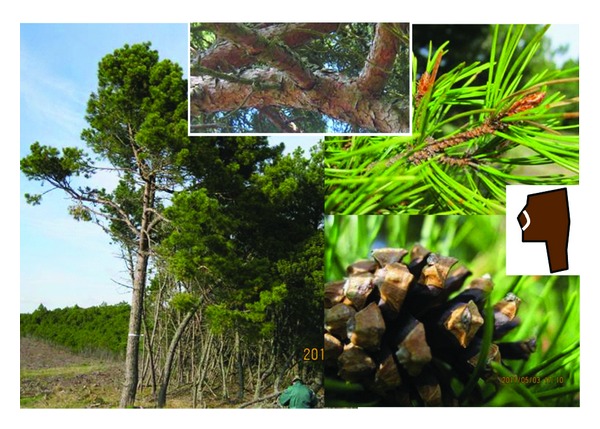
Putative *P. mugo *(female) ×* P. sylvestris* (male) hybrid (H S × M, group B in Figure 5, Table 2, ID PD56). Left, monocormic, needles fresh green (*mugo*, note pure *P. mugo* trees in the background and to the right with the same fresh green needle colour), upper stem with dark brown flakes (*sylvestris*), no paper flakes (*mugo*); upper right: shoots dark green brown glossy (*mugo*), needles fresh green (*mugo*), stem unit tip does not bend backwards at the needle sack (sylvestris); lower right: cone apophysis glossy (*mugo*) medium-thick hook (intermediate), umbo bordered (*mugo*), rather symmetric (*sylvestris*).

Group B contained mainly adult individuals with fresh green needles like typical *P. mugo* but brown bark and close to straw-coloured shoots, flat-to-medium hooked cone apophysis, and other traits more common to *P. sylvestris* (Figure 4). The WSS index of the group B was markedly lower than that of group A, meaning that the group B was morphologically closer to *P. mugo *(Table 3). Also a difference was noted in the presence of a dark-coloured border around the umbo of the cone apophysis: *P. sylvestris* has a borderless umbo and *P. mugo* a bordered umbo.

### 3.2. Needle Micromorphology

Averaged overall 470 trees investigated, the number of stomata rows on the abaxial (curved) and adaxial (flat) surfaces of the needle were 8.6 (stand. dev. = 2.1; min. = 4; max. = 15) and 7.9 (stand. dev. = 2.1; min. = 3; max. = 15), respectively. The overall mean number of stomata per 5 mm row was 53.4 (stand. dev. = 5.8; min. = 34; max. = 72).

**Figure 5:**
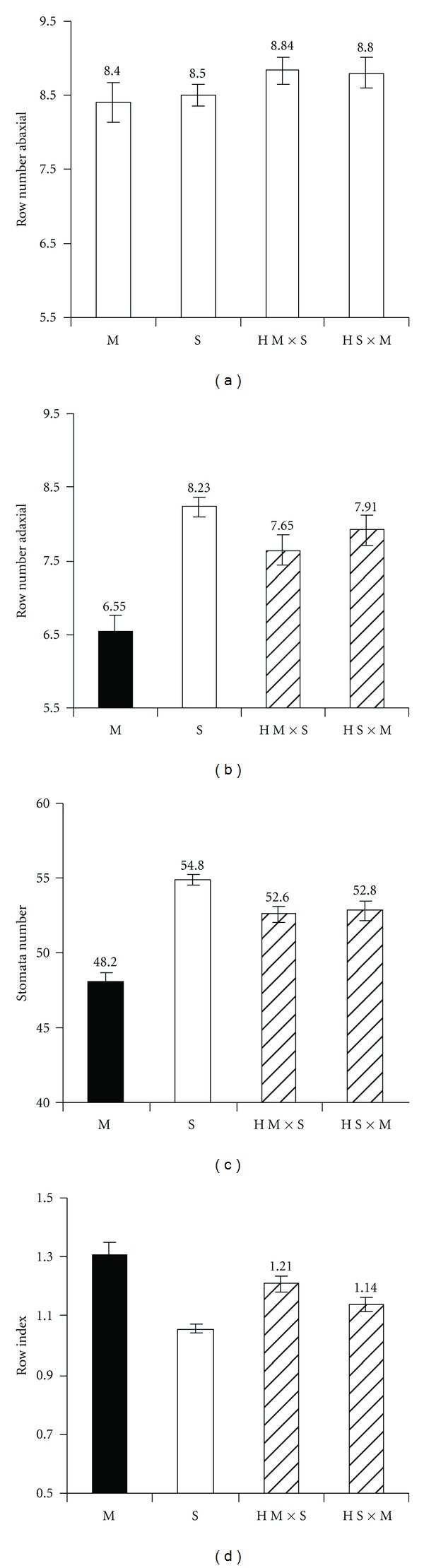
Species mean values for needle anatomy traits: stomata row number in the abaxial (a) and adaxial (b) needle surface; stomata number per 5 mm stomata row (c); index of stomata rows (abaxial/adaxial). The species which were significantly different at 0.05 *P* level are filled with different pattern (based on Tukey LSD test in ANOVA). M is *P. mugo* (40 needles from 4 trees were studied); S *P. sylvestris* (240 needles from 24 trees); H M × S putative hybrids *P. mugo *×* P. sylvestris* (group B in Table 2; 110 needles from 11 trees studied); H S × M hybrids *P. sylvestris *×* P. mugo* (group A; 80 needles from 8 trees). The error lines indelicate the standard errors (calculated from needle level). Mean values are given above the bars.

The ANOVA revealed significant effects of both species and tree within the species on all the needle micro-morphology traits studied (Table 4). The significance of the species effect was the lowest for the stomata row number on the abaxial (curved) needle surface (Table 4). The putative hybrids possessed a significantly higher number of stomata rows on the adaxial (curved) needle surface than both *P. sylvestris *and* P. mugo*, between which the difference was not significant (Figure 5(a)). Interestingly, the stomata row number on the adaxial (flat) surface was the lowest for *P. mugo* (6.5) and the greatest for *P. sylvestris *(8.2). The two groups of hybrids exhibited significantly different intermediate values; that is, the hybrid group with *P. mugo* as female was closer to *P. mugo* and hybrid group with *P. sylvestris* as the female was closer to *P. sylvestris* (Figure 5(b)). The stomata row index amplified the species differentiation described above: contrasting indexes of *P. mugo* (high) and *P. sylvestris* (low) with intermediate index values for the putative hybrids being closer to the species of the female parent (Figure 5(d)). *P. mugo* possessed the lowest (48) and *P. sylvestris* the highest (55) number of stomata per 5 mm row interval (Figure 5(c)). Again, the hybrids were intermediate between the *P. sylvestris *and* P. mugo* stomata numbers of 52.6 and 52.8 (Figure 5). For a dendrological reference, the descriptive statistics of the needle properties are given in Table 5.

Note that, for all the needle traits except the row number of abaxial surface, the differences between *P. mugo* and *P. sylvestris *as well as between hybrids and the parental species were significant at the 0.05 probability level (Figure 5). When “tree” was considered as a single source of variation in the ANOVA model, the significance level of the tree effect was rather uniform and the proportion of variation explained by the model was as high as 70% for all the needle micro-morphology traits (Table 4). Coefficient of variation for the stomata number was markedly less than that for the rest of the traits (Table 4).

The correlation analysis revealed the following (Table 6): the number of stomata rows on abaxial was not perfectly correlated with number of rows on adaxial surface (*r* = 0.63; *P* = 0.0001; *n* = 470); the correlation coefficient between adaxial (flat surface) row number and stomata number was positive and markedly higher than that for the abaxial (curved) surface (*r* = 0.09/*P* = 0.05 versus *r* = 0.22/*P* = 0.001; *n* = 470; Table 6). This difference was more pronounced at the tree mean level, where the correlation between abaxial row number and stomata number was not significant (Table 6).

The correlation coefficients calculated on needle level for each species separately revealed species-specific relationships between the number of stomata rows per needle and the stomata number per row: for *P. mugo* more stomata rows were associated with less stomata per row (*r* = −  0.17/n.s. and *r* = −51/*P* = 0.0008 for abaxial and adaxial; resp., *n* = 40), whereas, for *P. sylvestris*, more stomata rows were associated with more stomata per row (*r* = −0.18/*P* = 0.0058 and *r* = 0.34/*P* = 0.0001 for abaxial and adaxial; resp., *n* = 240). The putative hybrid material has correlations between the row number and stomata number which are intermediate to those of the parents, and they tend to be closer to the female parent: for H M × S, *r* = −0.22/*P* = 0.0232, and *r* = −0.22/*P* = 0.0181 for abaxial and adaxial, respectively, *n* = 110 (closer to *P. mugo*); for H S × M, *r* = 0.31/*P* = 0.0055 and *r* = 0.28/*P* = 0.0155 for abaxial and adaxial, respectively, *n* = 80 (closer to *P. sylvestris*).

## 4. Discussion

Phenotypically, the adult putative hybrids found in our study are estimated being at least 60 years old. This means that these trees germinated around 1950 and assuming the fact of the first *P. mugo *introductions date back to 1820 (*P. mugo* subsp. *rotundata *and subsp*. mugo*) and 1890 (*P. mugo *subsp*. uncinata*), they could well be both (a) naturally regenerated and spontaneous hybrids formed *in situ* and (b) hybrids born in Denmark and brought as seeds to the spit of Nerija. Assuming that *P. mugo* was introduced in 1820 and the sexual maturity time of *P. mugo *being 10 years, theoretically, there are at least 19 mating chances which could result in the formation of hybrids.

*P. sylvestris and P. mugo* are taxonomically close and according to [17] belong to the section *Pinus*, subsection *Sylvestres* Loud. In agreement with [18], flowering synchronization (*P. sylvestris* dispersing pollen earlier) between the two pine species in Nerija should not be a strong factor preventing hybridization because of (a) large microsite variation (dunes) creating warmer and colder niches, which cause large variation in flowering time among trees during one season (our observation, not presented here), and (b) the transfer effect of *P. sylvestris* introduced from various parts of Lithuania and may be Germany causing large variation in timing of flowering [19, 20]. In addition, the successful artificial hybridization experiments with *P. mugo *and* P. sylvestris* indicate that there are no biological barriers to fertilization and sound development of embryo, seedling, and tree [8, 21, 22]. All the above-described factors indicate a favourable environment for natural hybridization between *P. mugo *and* P. sylvestris* at the Lithuanian seaside.

However, the specific seaside environment and poor sandy soils may cause an unusual expression of certain traits in both pine species. First of these is the needle colour, which in case of a deficiency in nutrients, salinity, and photosynthesis rate may vary; see for example [23]. However, in such cases, the variation in discoloration differs between the parts of the crown, unless whole tree is seriously damaged. In our survey, no marked needle colour variation was observed between different parts of the crown of the individuals selected based on an unusual needle colour. Consequently, the probability of environmental error in needle colour as an indicator trait is low. Shoot colour depends on its physiological stage during the season. However, if the shoots of the last year are assessed, then this bias is eliminated and shoot colour can be used as an indicator [5]. Bark colour is a good indicator of *P. sylvestris*, as all adult pure *P. sylvestris* seen by us bear the light brown bark with papery flakes at the upper part of stem. Another indicator not affected by the microenvironment is the shape of the cone apophysis, where a hooked apophysis is a good indicator of *P. mugo *subsp.* uncinata* ([13], Figure 2) and some forms of *P. sylvestris* [5]. In our study, the backwards bending shoot bark tips at the stem units on shoots (Figure 2(b)) were attributable exclusively to *P. mugo* and to our knowledge not previously reported. It may be caused by a high density of needles on the shoots. However, owing to the absence of earlier scientific evidence on the importance of this trait, we gave it a low weight. To summarize, the probability of an environmental error in the WSS score used to distinguish species in our study is not high consequently, if an adult individual bears glaucous needles, cones with flat apophysis (feature common to *P. sylvestris* in Nerija), and dark bark without papery flakes together with the other traits more common for *P. mugo*, there is a high probability of the individual being a hybrid.

With regard to the needle micro-morphology, the stomata number and the stomata row index were found in our study to fall well within the range observed for both species in the earlier studies (e.g., the row index in [8] was 1.32 for *P. mugo* and 1.12 for *P. sylvestris*; in [15] it was 1.00 to 1.08 for *P. sylvestris*). However, the number of stomata rows on both needle surfaces may vary between the different studies. Thus, as a taxonomic indicator to be used for comparison among different studies, the index of stomata rows is a more desired criterion than the original numbers of stomata rows. In pines, the stomata number and stomata row number are dependent on the needle area, which in turn is influenced by the water availability [24, 25]. In milder climates, *P. sylvestris* needles tend to be bear more stomata than those in dryer windy areas, where the trees must restrain the transpiration surface to reduce the loss of water [26]. An intraspecific variation in stomata row number was observed in provenance tests, where the northerly origins possessed less stomata rows than those of more southerly origin [15]. The differential adaptive targets regarding wind and drought climate may also lead to the interspecific differences between *P. sylvestris* and *P. mugo, *the latter with less stomata, representing harsh climates. In *P. mugo*, the negative relationship between the stomata row number and the stomata number per row found in our study supports the preservationist approach of *P. mugo*—a surplus of stomata rows is “compensated” by fewer stomata per row.

We found two groups of putative hybrids, that is, group A with high and group B with low values of the WSS index (Table 2). Important common features of the individuals of group A were the greyish green needles and a shoot colour and bark type resembling those of *P. sylvestris *(Figure 3). The individuals of group B possessed the fresh green needles (a property of* P. mugo*) with the other properties of an intermediate character between *P. mugo* and *P. sylvestris* (Figure 4). Assuming that in *P. mugo* × *sylvestris *hybrids the maternal genotype has the greater effect on the morphology of the hybrid progeny [19, 20], it is likely that the female parent of the trees in group A was *P. sylvestris* and the female parent of the trees in group B was *P. mugo*. In an identification key to spontaneous *P*. × *rhaetica *and its parents in the Alps and Pyrenees, [5] stresses the following traits: luster of cone, bordered umbo, needles greyish green, bark splitting in angular dark brown plates. The authors in [8] morphologically described the spontaneous hybrids found in naturalized mixed stands of* P. mugo *and* P. sylvestris* in Denmark as follows: occasionally a shrub, greyish green needles, shoots ochre-coloured (light yellow brown), bark greyish brown, cones with moderately to strongly hooked apophyses, and greyish, bordered umbo. In our study, the above-listed properties resemble the individuals of group A more than those of group B. In none of the studies describing the morphology of the *P. mugo *subsp*. uncinata*, the brown fissured bark at the upper stem or greyish-green needles of *P. mugo* subsp. *uncinata* were reported [4, 13, 16, 27–32].

The variation in micromorphological traits of the needles also lend support for the hypothesis of hybridization. Both hybrid groups A and B had intermediate needle traits differing significantly different from those of the parental species (Figure 5). Furthermore, the individuals ascribed to the hybrid group A (hybrids with *P. sylvestris* as the female, H S × M) possessed a lower index of stomata rows than the hybrid group B (*P. mugo *the female parent, H M × S) and so were closer to their female parent's index value (Figure 5). Similarly, a closer proximity to the stomata row index of the female parent was observed for the hybrid group B (H M x M, in Figure 5). The correlation analysis reflects similar trends of this hybrid group differentiation. Like *P. mugo*, the individuals of hybrid group B (*P. mugo* as female parent) tended to have negative correlations between the row numbers and stomata number, whereas the individuals of the hybrid group A exhibited positive correlations, similar to pure *P. sylvestris*.

Thus, the patterns of variation found in morphology and needle micro-morphology among the individuals of *P. mugo*, *P. sylvestris*, and their putative hybrid provide a strong support for the hypothesis of spontaneous hybridization in Nerija. The variation of cone properties may depend on which of the *P. mugo* subspecies is involved in the hybridization: subsp. *uncinata* bears cones with hooked apophyses and subsp. *rotundata* has intermediate cones, while subsp. *mugo* has cones with flat apophyses [5]. The authors in [8] noted that *P. *×* rhaetica *was morphologically closer to *P. sylvestris* than to *P. mugo*. In the area around Juodkrantė, most hybrids were found in proximity of *P. mugo* subsp. *uncinata.* Here there are more individuals of *P. mugo* subsp. *uncinata* than in the other areas surveyed. It may be hypothesized that the formation of hybrids is more successful when *P. mugo* subsp. *uncinata* is one of the parents of *P*. x *rhaetica*. In fact, *P. mugo* subsp. *uncinata* may have become erect and monocormic as a result of the incorporation of *P. sylvestris* genetic material in its ancient past [33].

Are hybrids identifiable on the basis of morphology alone? The variation of the key traits in Table 3 and Figure 5 apparently exceeds the limits of environmental modifications. The hybrid group A, *P. sylvestris* (female) × *P. mugo* (male) is recognized by (Table 3): brown bark of the upper stem and blue green needles (properties of *P. sylvestris*) combined with cones having hooked and shiny apophyses and the absence of straw-coloured shoots, as well as the bark not peeling off in papery flakes (properties of *P. mugo*) (Figure 3), whereas the group B (*P. mugo* x *P. sylvestris* in Table 3) exhibited *P. mugo* type needles (fresh green) and cones but bark and shoot properties resembling *P. sylvestris* (Figure 4). The-above described combinations of traits exceed the intra-species variation limits and are strong indicators of spontaneous hybridization.

In conclusion, we found individuals possessing mixed morphology of both *P. mugo* and *P. sylvestris* in the same individual, which provides a strong evidence of spontaneous hybridization between* P. mugo *and* P. sylvestris* in the Lithuanian seaside spit of Kursiu Nerija. The putative hybrids cluster into two groups with the mixed morphology closer to the species of the putative female parent.

## Figures and Tables

**Table 1:** The localities and number of sampled putative *P. mugo *×* sylvestris *hybrids at each locality in Kursiu Nerija. Altitude of the localities varied from 5 to 20 m. a.s.l.. For the sampling interval, the geographical coordinates indicate the midpoint of the interval. The localities follow the order towards the south (Figure 1).

No.	Locality	Tree number	Latitude	Longitude	Sampling interval
(1)	Smiltinė	23	55° 38′	21° 06′	5 km along the seaside dune: up to border of Juodkrantė forest district
(2)	Juodkrantė	69	55° 30′	21° 06′	*P. mugo* stand 5 km northwards from Juodkrantė
(3)	Dvirači*ų* takas Juodkrantė-Smiltynė	49	55° 35′	21° 07′	10 km interval northwards from Juodkrantė
(4)	Pervalka	46	55° 25′	21° 05′	5 km interval from the Reza monument to the Naglis reserve
(5)	Voice Krugas	10	55° 21′	21° 02′	Dune of Voice Krugas (midway Nida to Preila)
(6)	Nida	6	55° 17′	20° 58′	At the foot of the Parnidžio dune

	Total	203			

**Table 2:** Key-traits separating *P. mugo *spp*. uncinata* and *P. sylvestris*. An intermediate value of a trait was scored as 0.5. The weights were used in calculating of weighted score sum (WSS).

Trait (abbreviation)	WSS weight adult	WSS weight young	Typical *P. mugo *subsp*. uncinata *	Typical *P. sylvestris *
			Score = 0	Score = 1

(1) Bark of the upper stem and main branches (BARK)	0.2	0.1	Fissured (small hard shells); grayish black	Peeling off in thin reddish brown flakes; dark brawn
(2) Needle colour (NEEDLE)	0.3	0.4	Fresh green	Blue green (glaucous)
(3) Cone apophysis (APOPH)	0.1	—	Hooked; blackish brown to brown; shiny	Flat; straw; dull;
(4) UMBO	0.05	—	Bordered by black or gray zone	Not bordered
(5) Symmetric cones (CONSYM)	0.05	—	No	Yes
(6) Tip of stem unit scale on the shoot bends backwards (TIPSU)	0.05	0.2	Yes	No
(6) Shoots of the previous year (SHOOT)	0.2	0.2	Grayish black; dark gray	Straw brawn
(7) Needle density on shoot (NDENSE)	0.05	0.1	High	Low
Needle anatomy (not used in WSS)				
Index of stomata rows (ROWINDEX)	—	—	High	Low
Number of stomata (STOMEAN) (mean of the estimates in two rows)	—	—	Low	High

**Table 3:** Representatives of putative *P. sylvestris *×* P. mugo* hybrids grouped into two groups according to the WSS value. The WSS weights sum up to 1. Pure *P. sylvestris* or *P. mugo* subsp. *uncinata* WSS indexes add up to 1 and 0, respectively (based on our observation; not shown). Number at the tree ID indicates the tree age (at least, based on phenotype). Trait abbreviations are explained in Table 1.

Tree ID	BARK	NEEDLE	APOPH	UMBO	CONSYM	TIPSU	SHOOT	NDENSE	WSS	H	D
Weight adult	0.2	0.3	0.1	0.05	0.05	0.05	0.2	0.05	1		
Weight young	0.1	0.4	—	—	—	0.2	0.2	0.1	1		
Min. value = 0	Grey	Green	Hook	Unbord.	Asym.	Yes	Grey	Dense	0-*uncinata *		
Max. value = 1	Brown	Grey	Flat	Border	Sym.	No	Straw	Sparse	1-*sylvestris *		
PD43 (60)	0.5	1	1	0.5	1	1	0.5	1	**0.78**	9	25
KN10 (60)	0.5	1	0	1	1	0.5	0.5	1	**0.68**	15	25
PR51 (40)	1	0	1	0.5	1	1	1	1	**0.68**	10	18
PAV12 (20)	0.5	1	0.5	0.5	1	0.5	0.5	1	**0.70**	10	10
KD43 (10)	0.5	1	0.3	0	0.5	0	1	1	**0.71**	2	4
KD59 (10)	0.5	1	0.5	0	0.5	1	0.5	1	**0.68**	2	4
PAV10 (15)	0.5	1	—	—	—	1	0	1	**0.75**	2	4
KD54 (4)	0.5	1	—	—	—	1	0.5	1	**0.85**	1	2
KD55 (6)	0.5	1	—	—	—	1	0.5	1	**0.85**	1	2
PD58 (5)	0.5	1	—	—	—	1	0.5	1	**0.85**	0	2
PAV8 (8)	0.5	1	—	—	—	1	0.5	1	**0.85**	1	3
PAV46 (5)	0.5	1	—	—	—	1	0.5	1	**0.85**	1	2
PD60 (5)	0.5	1	—	—	—	1	0.5	1	**0.85**	0	2
Mean group A (H S × M)^1^	**0.54**	**0.92**	**0.55**	**0.42**	**0.83**	**0.85**	**0.54**	**1.00**	**0.77**		

KD48 (60)	0.5	0	0.5	1	0.5	0.5	0.5	1	**0.40**	15	30
KR43 (60)	0.5	0	0.5	0	0.5	0.5	0.5	0.5	**0.33**	4	15
KD57 (60)	1	0	0.5	0.5	0.5	0	1	0	**0.50**	12	25
KD56 (60)	1	0	0	0	0	0.5	0.5	0.5	**0.35**	11	25
KD51 (60)	0.5	0	0.5	0.5	0.5	0	1	0	**0.40**	10	30
KD58 (60)	1	0	0.5	0.5	0.5	1	0	0.5	**0.38**	12	25
KAV7 (60)	0.5	0.5	0.5	0.5	0	1	0	1	**0.43**	10	25
KAV50 (60)	0.5	0.5	0.5	0.5	0	1	0	1	**0.43**	8	18
PD8 (60)	0.5	0	0.5	0.5	0.5	0.5	0.5	1	**0.40**	18	20
KD49 (5)	0.5	0.5				1	0	1	**0.35**	0	3
Mean group B (H M × S)^1^	**0.67**	**0.17**	**0.44**	**0.44**	**0.31**	**0.61**	**0.39**	**0.61**	**0.39**		

H M × S abbreviates hybrids* mugo × sylvestris*, and H S × M vice versa.

**Table 4:** Results of the ANOVA on the needle traits according the two models: No. 1 with tree as a single effect and No. 2 with the effects of species and tree nested within SPECIES in the model. DF is degrees of freedom. R^2^ is proportion of variance explained by the model. CV is the coefficient of variation.

Source	Row number abaxial (curved side)		Row number adaxial (flat side)		Mean stomata number (flat side)		Stomata row index	
	*F*	Pr > *F*	*F*	Pr > *F*	*F*	Pr > *F*	*F*	Pr > *F*
		Model 1 (DF tree is 46; DF ERROR is 423)						

TREE	21	0.0001	20	0.0001	21	0.0001	5.0	0.0001
R^2^(CV%)	0.69	(14)	0.68	(16)	0.69	(6)	0.35	(20)

		Model 2 (DF SPECIES is 3; DF tree (SPECIES) is 43; DF ERROR is 423)						

SPECIES	3	0.036	23	0.0001	50	0.0001	21	0.0001
Tree (SPECIES)	22	0.0001	19	0.0001	18	0.0001	4	0.0001
R^2^	0.70		0.70		0.70		0.4	

**Table 5:** The species means and standard errors (SE) for the needle traits. *P. mugo*: 40 needles from 4 trees were studied; *P. sylvestris:* 240 needles from 24 trees; H M × S: putative hybrids *P. mugo *×* P. sylvestris* (group B in Table 2): 110 needles from 11 trees; H S × M: hybrids *P. sylvestris *×* P. mugo* (group A; 80 needles from 8 trees).

Species	ROwABAX	*Se*	ROWADAX	*Se*	STOMR1	STOMR2	STOMEAN	*Se*	ROWINDEX	*Se*
*P. mugo*	8.4	*0.27*	6.6	*0.20*	47.5	48.8	48.2	*0.5*	1.31	*0.04*
*P. sylvestris*	8.5	*0.15*	8.2	*0.14*	54.6	55.1	54.8	*0.4*	1.06	*0.02*
H S × M (group A)	8.8	*0.21*	7.9	*0.21*	52.9	52.8	52.8	*0.7*	1.14	*0.02*
H M × S (group B)	8.8	*0.18*	7.6	*0.22*	52.4	52.7	52.6	*0.5*	1.21	*0.03*

ROWABAX and ROWADAX-number of stomata rows on abaxial (curvy) and adaxial (flat) needle surface; STOMR1 and STOMR2: number of stomata per 5 mm interval in row 1 and row 2; STOMEAN: mean of STOMR1 and STOMR2; ROWINDEX: stomata row index = ROWABAX/ROWADAX.

**Table 6:** Pearsons product-moment correlation coefficients among the needle traits calculated on the tree mean level (upper diagonal) and the individual needle level (lower diagonal). The significance level and number of observations are given below the coefficients.

Trait	Row no. abaxial (flat)	Row no. adaxial (curved)	Stomata number in row 1	Stomata number in row 2	Mean stomata number	Stomata row index
Row no. abaxial (flat)	1	**0.80**	**0.09**	**0.09**	**0.09**	**0.16**
	0	0.0001	0.5696	0.5664	0.5632	0.2875
		47	47	47	47	47

Row no. adaxial (curved)	0.63	1	**0.36**	**0.32**	**0.34**	**−0.45**
	0.0001	0	0.0128	0.03	0.0181	0.0015
	470		47	47	47	47

Stomata in number row 1	0.09	**0.22**	1	**0.95**	**0.99**	**−0.44**
	0.0547	0.0001	0	0.0001	0.0001	0.0021
	470	470		47	47	47

Stomata number in row 2	0.08	**0.19**	**0.74**	1	**0.99**	**−0.38**
	0.0731	0.0001	0.0001	0	0.0001	0.0092
	470	470	470		47	47

Mean stomata number	0.09	**0.22**	**0.94**	**0.93**	1	**−0.41**
	0.0464	0.0001	0.0001	0.0001	0	0.0039
	470	470	470	470		47

Stomata row index	0.32	**−0.51**	**−0.15**	**−0.13**	**−0.15**	1
	0.0001	0.0001	0.0008	0.0056	0.001	0
	470	470	470	470	470	
